# Kyphoscoliosis: clinical image

**DOI:** 10.11604/pamj.2023.44.64.38840

**Published:** 2023-02-01

**Authors:** Pallavi Dhulse, Bibin Kurian

**Affiliations:** 1 Department of Child Health Nursing, Srimati Radhikabai Meghe Memorial College of Nursing, Datta Meghe Institute of Higher Education and Research, Sawangi (Meghe), Wardha, Maharashtra, India

**Keywords:** Deformity, spinal cord, thoracolumbar spine, kyphoscoliosis

## Image in medicine

The term “kyphoscoliosis” refers to a deviation from the spine's typical curvature in the sagittal and coronal planes, which may also involve a rotation of the spinal axis. A lateral deviation of more than 10 degrees in the coronal plane, as determined by the Cobb angle, is considered to be an adult case of scoliosis. Postural variation is responsible for lateral variations of fewer than 10 degrees. The terms kyphosis and lordosis describe how the spine curves in the sagittal plane. When the spine is seen laterally, the cervical and lumbar spines both exhibit a degree of lordosis (posterior curvature) that typically ranges between 35 and 80 degrees, while the thoracic spine exhibits a degree of kyphosis (forward curvature) that typically ranges between 30 and 50 degrees. About 1 in 1000 people have the spinal ailment kyphoscoliosis, and about 1 in 10,000 of those people have a severe spinal deformity. A 16-year-old girl complained of deformity in spine, difficulty in breathing, pain, weakness deformity increases significantly and becoming worse was taken to the outpatient clinic. After collection of medical history and physical examination revealed that she had kyphoscoliosis from 2 years ago and it increases significantly. Her X-ray also revealed that she is having kyphoscoliosis, and thus the doctor referred them to the inpatient unit for additional surgical treatment. Example of disease where kyphoscoliosis can occur include vertebral fracture, Scheuermann disease, adolescent idiopathic scoliosis.

**Figure 1:**
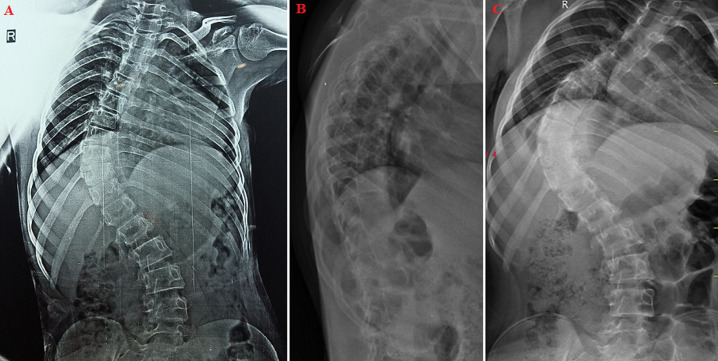
A, B, C) X-ray presentation of kyphoscoliosis

